# From Molecule to Meaning: Neuronopathic Biomarkers and Clinical Relevance in GM1

**DOI:** 10.1002/jimd.70134

**Published:** 2026-01-07

**Authors:** Krista Casazza, Roberto Giugliani, Debra S. Regier, Jeanine Jarnes

**Affiliations:** ^1^ Purity Health Franklin Tennessee USA; ^2^ DASA, Federal University of Rio Grande Do Sul (UFRGS), hospital de Clinicas de Porto Alegre (HCPA) Porto Alegre Brazil; ^3^ Children's National Rare Disease Institute, Children's National Hospital Washington District of Columbia USA; ^4^ Department of Pediatrics University of Minnesota Medical School Minneapolis Minnesota USA; ^5^ Pharmacotherapy for Inherited Metabolic Diseases, Advanced Therapies Department College of Pharmacy, Experimental and Clinical Pharmacology Minneapolis Minnesota USA

## Abstract

GM1 gangliosidosis is a rare, progressively neurodegenerative lysosomal storage disorder characterized by profound central nervous system involvement and substantial clinical heterogeneity. The development of reliable biomarkers is essential for tracking disease progression, stratifying patients, and advancing clinical trial readiness. Primary substrate markers, including cerebrospinal fluid (CSF) GM1 ganglioside and related lysosphingolipids, provide direct biochemical indices of lysosomal dysfunction. Emerging glycan signatures may further reflect disruptions in glycosylation pathways and responses to therapeutic intervention. Neurofilament light chain (NfL), a sensitive indicator of axonal injury, is consistently elevated in GM1 and shows promise as a fluid biomarker, although it does not convey regional specificity. Neuroimaging offers complementary insight into the structural and metabolic consequences of disease. Characteristic findings include diffuse white matter abnormalities, thalamic and basal ganglia signal changes, cortical and cerebellar atrophy, and ventriculomegaly. Quantitative MRI and magnetic resonance spectroscopy (MRS) reveal longitudinal declines in tissue volume and neuronal integrity that parallel functional deterioration. Diffusion‐based methods, including differential tractography, highlight progressive loss of white matter microstructure relative to age‐matched controls. However, severe anatomical distortion in GM1 limits the applicability of standard neuroimaging atlases, necessitating manual or semi‐automated segmentation approaches. Together, fluid biomarkers (gangliosides, lysosphingolipids, glycans, NfL) and advanced neuroimaging metrics (volumetry, MRS, diffusion imaging) establish a multimodal framework for evaluating disease burden and therapeutic response. Standardized methodologies, harmonized natural history datasets, and genotype‐stratified analyses will be critical for validating these biomarkers across GM1 subtypes. Strengthening this biomarker ecosystem will enable sensitive and clinically meaningful endpoints to support future therapeutic development in GM1 gangliosidosis.

## 1 Setting the Stage—The Current Landscape

GM1‐ganglioside, a major glycosphingolipid (GSL) of the vertebrate central nervous system (CNS), plays key roles in neuronal function. Although the distribution pattern and lipid composition are dependent upon cell and tissue type and developmental stage, over 80% of the glycocalyx of brain cells are GSL [1]. Thus, the broad spectrum of functions of GM1‐ganglioside in various neurological and neuropathological processes is well established. GM1‐gangliosidosis is caused by a deficiency in the enzyme β‐galactosidase, which leads to the accumulation of GM1‐ganglioside in the brain, liver, and other organs [2]. The pathogenic pathway of GM1‐gangliosidosis storage involves both the accumulation of toxic products causing inflammatory response and of mitochondrial abnormalities, leading to cell death with the CNS predominantly affected [2]. The estimated incidence of GM1‐gangiosidosis is 1:100 000–200 000 live births, with a greater prevalence in specific geographical areas (Brazil, Japan, Malta) [3].

## 2 Clinical Nomenclature

GM1‐gangliosidosis phenotypes are primarily differentiated by age of onset and developmental milestones [4, 5]. Four clinical phenotypes span a continuum from infantile to late‐infantile, juvenile, and adult onset [5]. The “severity” or rate of progression can be described as severe, with rapid progression, to more attenuated forms presenting later in life, often referred to as “adult” phenotypes. The earlier the age of clinical presentation, the more severe and rapid the clinical progression.

## 3 Type 1, Infantile GM1‐Gangliosidosis

Infantile GM1 (or Type I) represents the most severe form of the disease and typically manifests in the first few months of life with developmental delay, muscle weakness, seizures, and sometimes an enlarged liver and spleen. Death typically occurs by the age of 3 years, with the most common cause of death being respiratory failure secondary to aspiration pneumonia [5, 6].

Akar et al. [7] characterized the clinical and biochemical profile of 14 affected infants (10 males) with onset of symptoms between birth and 5 months of age. The median interval from symptom onset to diagnosis was 4.3 months (range: 0–13 months). The most common initial manifestation was motor delay. During a median follow‐up period of 14.8 months (range: 0.4–92.2 months), 12 patients died before 24 months of age, with a median survival time of 21.3 months. Importantly, higher residual leukocyte β‐galactosidase activity was associated with later age at onset, delayed diagnosis, and prolonged survival, underscoring the relationship between residual enzyme function and disease severity [7].

## 4 Type 2, Juvenile Forms of GM1‐Gangliosidosis

Type II GM1‐gangliosidosis encompasses two subtypes—late‐infantile (Type IIa) and juvenile (Type IIb)—which represent intermediate phenotypes between the severe infantile and the attenuated adult forms of the disease. In both subtypes, affected children typically achieve normal developmental milestones during the first year of life, distinguishing them from the infantile form characterized by onset in early infancy [4, 5, 6]. In Type 2a (late‐infantile), children began to show signs of developmental delay and loss of developmental skills previously gained, around the age of 1. Children with the late‐infantile subtype generally exhibit developmental plateau or regression around 12 months of age. This regression often includes loss of acquired motor and cognitive skills such as sitting, crawling, or expressive language. Neurological decline is accompanied by progressive hypotonia, seizures, and gait instability. As the disease advances, pyramidal and extrapyramidal signs emerge, reflecting widespread neurodegeneration [5, 6]. In the classic juvenile form, symptom onset typically occurs between 2 and 5 years of age, following a period of normal early development. The earliest signs are declining ambulation and speech deterioration, which progress gradually over several years. Unlike the infantile form, cherry‐red macular spots and hepatosplenomegaly are usually absent, and skeletal abnormalities are often subtle. The disease course is slowly progressive, leading to loss of independent ambulation and severe speech impairment over time, but survival may extend into late childhood or adolescence [4]. The juvenile phenotypes are clinically distinct from the infantile form by their slower rate of neurodegeneration and later onset of symptoms, though diagnostic overlap with other neurodegenerative or ataxic syndromes is common. The absence of visceral involvement and characteristic ophthalmologic findings frequently delays recognition, resulting in misdiagnosis as other neurological or metabolic conditions [4, 5, 6]. For instance, Karimzadeh et al. [8] reported a case initially misdiagnosed as juvenile idiopathic arthritis, later correctly identified as late‐infantile GM1‐gangliosidosis through β‐galactosidase enzyme assay and *GLB1* genetic testing, prompted by radiographic evidence of dysostosis multiplex and progressive neurological regression. This case underscores the necessity of considering lysosomal storage disorders in the differential diagnosis of unexplained neurodegeneration or skeletal dysplasia in early childhood.

## 5 Type 3, Late‐Onset of Adult‐Onset GM1‐Gangliosidosis

Type III, or adult‐onset GM1‐gangliosidosis, represents the mildest and slowest‐progressing phenotype within the GM1 disease spectrum. In contrast to the infantile and juvenile forms, only individuals with Type III GM1‐gangliosidosis typically survive into adulthood. Clinical onset often occurs during late childhood, adolescence, or early adulthood, and disease progression spans several decades [9]. Half of the reported families with type 3 GM1‐gangiosidosis come from Japan with almost all harboring a homozygous pathogenic founder p. (Ile51Thr) variant [6].

## 6 Importance of Distinguishing Phenotypes While Acknowledging the Disease Spectrum

More than 90% of adult‐onset GM1 patients develop movement disorders, with generalized dystonia as the predominant motor manifestation throughout the disease course. Additional extrapyramidal features, including akinetic‐rigid parkinsonism, are frequently observed, particularly, among individuals presenting with short stature or mild skeletal dysplasia. Tremor, bradykinesia, and rigidity may mimic idiopathic Parkinson's disease, often leading to misdiagnosis or delayed recognition. Cognitive decline, when present, is usually mild and evolves slowly compared to earlier‐onset subtypes [8, 10]. Although historically classified into four clinical subtypes (infantile, late‐infantile, juvenile, and adult), GM1‐gangliosidosis is increasingly recognized as a continuum of phenotypic severity rather than distinct categories. A study employing whole‐exome sequencing (WES) in four children presenting around age 3 years identified rare pathogenic *GLB1* missense variants associated with ataxia, dystonia, and global developmental regression. These findings support the concept that genotypic diversity drives a continuous clinical spectrum, bridging phenotypes previously considered discrete. Recognizing this overlap is critical for accurate diagnosis, genotype–phenotype correlation, and therapeutic stratification [10].

While it is essential to appreciate the overlapping features across subtypes, phenotypic differentiation retains significant clinical and translational value. Stratification by subtype enables the identification of appropriate clinical trial endpoints tailored to disease stage and rate of progression. In rare neurodegenerative conditions such as GM1‐gangliosidosis, selecting outcome measures that align with the temporal pattern of neurological decline (e.g., motor, cognitive, and biomarker‐based endpoints) is fundamental to improving trial design, statistical power, and interpretability. Despite advances in elucidating GM1 pathophysiology and establishing robust animal and cellular models, no globally approved disease‐modifying therapy currently exists. Management remains confined to supportive and palliative interventions aimed at alleviating neurological and systemic complications. The pronounced clinical heterogeneity across subtypes, combined with the high disease burden in early‐onset forms, highlights the urgent need for reliable biomarkers to facilitate earlier diagnosis, monitor disease progression, and assess therapeutic efficacy. Biomarker‐driven approaches are expected to accelerate drug development and regulatory approval in this rare, life‐limiting disorder [4, 5, 6].

## 7 Lack of Effective Treatments for GM1‐Gangliosidosis

Despite substantial advances in understanding the molecular pathophysiology of GM1‐gangliosidosis and the development of several robust animal and cellular models, no disease‐modifying therapy has yet received regulatory approval worldwide. Current clinical management remains limited to supportive and palliative interventions, aimed at symptom relief, respiratory management, nutritional support, and prevention of secondary complications. The marked phenotypic heterogeneity across subtypes—ranging from the rapidly progressive infantile form to the slowly evolving adult‐onset variant—poses significant challenges for therapeutic development. The severe and early‐onset forms (particularly, Type I) are associated with profound neurodegeneration, systemic involvement, and limited survival, resulting in substantial disease burden for patients and families. Consequently, there is a critical need to establish validated biomarkers that can [1] enable earlier diagnosis [2] stratify disease severity [3] guide clinical trial design, and [4] monitor therapeutic response. Such biomarkers are pivotal to accelerating translational research, facilitating regulatory pathway alignment, and improving genotype‐specific counseling and care planning [6, 11].

## 8 Animal Models

Animal models have been instrumental in elucidating disease mechanisms, identifying biomarkers, and advancing therapeutic strategies for GM1‐gangliosidosis. Currently, five murine models have been reported, generated using different *Glb1* gene‐targeting strategies. These models vary in the age of onset, biochemical phenotype, and lifespan, yet collectively recapitulate the neuropathological hallmarks of severe human GM1 disease, including lysosomal GM1 accumulation, neuronal vacuolation, demyelination, and gliosis. Although limited by differences in brain size and lifespan, murine models remain indispensable for mechanistic studies, early biomarker validation, and preliminary therapeutic screening [6]. The naturally occurring feline model of GM1‐gangliosidosis has proven especially valuable due to its close recapitulation of human infantile disease. Cats harboring *GLB1* loss‐of‐function variants exhibit progressive neurodegeneration, motor dysfunction, and lysosomal storage pathology nearly identical to the human phenotype. This model has provided critical insights into disease progression and biomarker kinetics and has served as a cornerstone for evaluating therapeutic modalities, including AAV‐mediated gene therapy, enzyme replacement, and substrate reduction approaches. Notably, AAV‐based intracerebral and systemic gene therapy studies in GM1 cats have demonstrated sustained enzymatic correction, delayed disease onset, improved motor function, and extended survival, establishing a strong translational precedent for human clinical trial [6, 12]. Similarly, canine GM1‐gangliosidosis, caused by naturally occurring *GLB1* variants in specific breeds (e.g., Shiba Inu, mixed breeds), has provided a large‐animal platform for translational research. Affected dogs display progressive ataxia, motor and visual dysfunction, and cognitive decline, paralleling the clinical trajectory observed in human patients [13, 14, 15, 16]. Neuropathological evaluation reveals extensive neuronal storage, demyelination, and astrocytosis, aligning closely with human histopathology. Importantly, AAV‐mediated gene therapy in GM1‐affected dogs has yielded robust therapeutic responses, including delayed symptom onset, improved neurological function, and markedly prolonged survival [17]. These findings not only validate the disease's mechanistic fidelity in large animals but also provide essential safety and efficacy data to support first‐in‐human trials [11, 18]. Together, murine, feline, and canine GM1‐gangliosidosis models constitute a translational continuum for bridging basic science with clinical application. These systems enable investigation of molecular pathogenesis, longitudinal biomarker dynamics, and therapeutic dose optimization under controlled conditions. Data generated from large‐animal models have been directly leveraged to inform vector design, delivery routes, and endpoint selection for ongoing human AAV‐based gene therapy trials. While preclinical evidence forms the foundation for biomarker discovery, the translation of nonclinical biomarkers to clinical endpoints remains a crucial next step. Integration of animal model data with human longitudinal studies will refine the identification of predictive and pharmacodynamic biomarkers, improve trial efficiency, and ultimately expedite therapeutic development for this devastating lysosomal disease.

## 9 Primary Objective

The primary objective of this review is to present a comprehensive synthesis of current evidence on biomarkers associated with GM1‐gangliosidosis, emphasizing their biological relevance, clinical applicability, and position within the causal disease pathway. By integrating findings from preclinical and clinical studies, the review delineates how specific biomarkers reflect underlying pathophysiological processes (i.e., lysosomal dysfunction, neuronal injury, and neuroinflammation) thereby providing mechanistic insight into disease progression and potential therapeutic response. In addition, this review critically examines the methodological rigor of biomarker research in GM1, with particular focus on:

- The biological matrix used for measurement (e.g., plasma, cerebrospinal fluid, urine, or tissue).
- The assay platforms and analytical validation frameworks employed to ensure reliability, reproducibility, and regulatory acceptability.
- The translational relevance of these approaches for clinical application.

Finally, the review evaluates the strength of evidence supporting each biomarker's utility across key contexts of use, including diagnostic, prognostic, disease‐severity, and therapeutic outcome measures. By consolidating these data, the review aims to identify existing knowledge gaps, highlight opportunities for biomarker standardization, and establish a foundation for regulatory and clinical qualification of biomarkers in GM1‐gangliosidosis.

## 10 Methods

### 10.1 Search Criteria

To provide a robust overview of current and emerging neuronopathic biomarkers in GM1, identifying biomarker candidates with clinical evidence to inform strategies for incorporating biomarkers with sufficient data into research and therapeutic development for GM1. First, clinical trial registries (ClinicalTrials.gov, EU Clinical Trials Register) were interrogated to capture completed, terminated, withdrawn, ongoing (recruiting, active not recruiting) trials identifying 16 trials in GM1‐gangliosidosis (Appendix A). Ideally, multiple data sets will allow for the identification of common data elements across biomarkers for more than one disease at a time. Next, a comprehensive search across scientific databases such as PubMed, Embase, and Scopus for peer‐reviewed articles on GM1‐gangliosidosis and related neurodegenerative conditions was conducted. Targeted keywords: “GM1,” “GM1‐gangliosidosis,” “GM1‐gangiosidosis biomarkers,” “neuronopathic biomarkers and GM1,” “neurodegeneration,” “lysosomal storage disorder biomarkers,” “lysosomal disease biomarkers.” The comprehensive approach to biomarker identification also evaluated neuroimaging as a potential biomarker, as such, neuroimaging techniques used to visualize brain pathology linked to GM1‐gangiosidosis were included in the search terms: “GM1‐gangiosidosis and brain atrophy, gray and white matter changes,” “Neimann–Pick and brain atrophy, gray and white matter changes,” “GM1‐gangiosidosis and neuronal loss, “GM1‐gangiosidosis neuroimaging,” “GM1‐gangiosidosis and brain atrophy,” and “GM1‐gangliosidosis and neuronal loss.” Subsequent searches in databases and clinical trials registry included biochemical markers such as proteins, lipids, metabolites, and nucleic acids that may reflect lysosomal dysfunction or neurodegeneration in GM1. Since GM1‐gangiosidosis shares pathophysiological similarities with other neurodegenerative diseases, biomarkers identified in related disorders were also included in search: “GM1‐gangiosidosis and GM2,” “GM1‐gangiosidosis and Neiman‐Pick,” “GM1‐gangiosidosis and lysosomal storage diseases.”

### 10.2 Clinical Evidence for Diagnostic Biomarkers in GM1‐Gangliosidosis

GM1‐gangliosidosis is a progressive lysosomal storage disorder caused by biallelic pathogenic variants in the *GLB1*, which encodes the enzyme β‐gal. This lysosomal hydrolase catalyzes the degradation of GM1 ganglioside and related glycoconjugates. Loss or reduction of β‐gal activity results in the progressive accumulation of GM1 ganglioside and its derivatives within lysosomes, most notably in the CNS but also in visceral tissues and biological fluids such as blood and urine [11] The pathological accumulation of GM1 ganglioside perturbs several critical cellular processes, including membrane trafficking, signal transduction, autophagic flux, and neuronal excitability. These disruptions collectively contribute to neuronal dysfunction, inflammation, and neurodegeneration, producing the clinical spectrum ranging from infantile to adult‐onset disease forms [6].

β‐galactosidase is synthesized as a precursor polypeptide that undergoes N‐linked glycosylation and proteolytic maturation before assembly into a functional lysosomal enzyme complex. Within lysosomes, β‐gal interacts with protective protein/cathepsin A (PPCA) and neuraminidase 1 (NEU1) to form a multienzyme complex essential for maintaining enzyme stability and activity. Disruption of this complex, whether through *GLB1* variants or secondary perturbations in PPCA or NEU1, leads to enzyme instability and impaired substrate degradation [3, 5, 6, 18, 19].

It is important to note that β‐galactosidase deficiency is observed also in two other lysosomal diseases: with secondary decreased activity of neuraminidase and beta‐galactosidase; (1) in Morquio syndrome type B disease (mucopolysaccharidosis type IVB), when the variant in the *GLB1* affects mainly the ability of β‐galactosidase to degrade keratan‐sulfate [20] and in (2) galactosialidosis, in this case secondary to pathogenic variants in the *PPCA* gene, leading to a deficient PPCA protein [20, 21]. Differentiating GM1‐gangliosidosis from these conditions requires combined enzyme activity profiling, substrate accumulation assays, and molecular genetic testing. This integrated diagnostic approach is vital for accurate classification, prognostic assessment, and eligibility determination for clinical trials targeting *GLB1*‐related disorders.

## 11 Diagnostic Biomarkers of GM1‐Gangliosidosis

Clinical diagnosis of GM1‐gangliosidosis relies primarily on biochemical assessment of β‐galactosidase activity, which is markedly reduced in affected individuals. Enzyme activity can be quantified using dried blood spots (DBSs), leukocytes, plasma/serum, or cultured fibroblasts. The degree of residual β‐gal activity correlates inversely with disease severity, lowest in the infantile subtype, intermediate in the juvenile form, and relatively higher in adult‐onset disease. Molecular confirmation is achieved through *GLB1* sequencing, which enables identification of pathogenic variants, genotype–phenotype correlation, and carrier detection for genetic counseling. Despite these established diagnostic modalities, early detection remains a major unmet clinical need, particularly, for asymptomatic or presymptomatic individuals who could benefit from future disease‐modifying interventions. Thus, identifying circulating or imaging biomarkers capable of detecting subclinical disease represents a critical translational objective and a potential outcome measure in forthcoming systemic therapy trials.

### 11.1 Newborn Screening (NBS)

Recent initiatives in NBS for GM1‐gangliosidosis leverage tandem mass spectrometry (MS/MS) and digital microfluidics to detect reduced β‐galactosidase activity. Liquid chromatography–tandem mass spectrometry (LC–MS/MS) offers the potential to directly quantify GM1‐ganglioside in DBSs [22, 23]. Multiplexing GM1‐gangliosidosis with other LDs is a growing trend, improving efficiency and cost‐effectiveness. Genetic approaches, particularly, next‐generation sequencing (NGS), are also gaining traction. Despite these advances, challenges remain. There are significant barriers related to optimizing sensitivity and specificity to minimize false positives, addressing ethical concerns around early diagnosis for conditions with limited treatment options, and establishing standardized follow‐up protocols. Measurement of enzymatic activity in DBS remains the preferred first‐tier NBS method. β‐galactosidase activity assays provide a direct diagnostic readout. Su et al. [23] demonstrated the utility of MS/MS‐based enzymatic assays in GM1 patients, identifying elevated glycan biomarkers (dp5 and A2G2) compared to unaffected controls. Herbst et al. emphasized the value of DBS assays but recommended second‐tier analysis using endogenous NRE biomarkers to reduce false positives [22].

While the development of diagnostic biomarkers for GM1‐gangliosidosis through NBS holds promise for early detection and represents a key focus for early diagnosis and intervention, in scenarios where NBS is not available or feasible, the focus shifts to identifying reliable diagnostic biomarkers for use later in the clinical course [4]. Elevated serum and CSF aspartate transaminase (AST) levels have been consistently reported in GM1, correlating with disease severity and age of onset. Infantile forms show clearer progression trends than late‐infantile cases, where variability complicates interpretation. Longitudinal studies suggest AST may serve as a monitoring biomarker [12, 18, 24]. Animal models reinforce this utility: GM1‐affected dogs and cats exhibit elevated AST that rises with disease progression, normalizing after AAV‐mediated gene therapy. These findings highlight AST as a candidate biomarker for both disease monitoring and therapeutic response. The recurrent variant, p.D448V in GLB1, accounted for 50.0% of total alleles, highlighting the need to better understand genotype–phenotype associations. Similarly, increased CSF AST levels have been observed in individuals with late‐infantile GM1‐gangiosidosis compared to those with the juvenile form of GM1‐gangiosidosis or healthy controls [25] Studies in GM1‐gangiosidosis animal models have explored CSF AST concentrations as markers of disease progression and treatment response. In GM1‐affected dogs, Satoh et al. [13] found baseline CSF AST levels were significantly higher than in normal dogs, rising with disease progression until plateauing around 7 months of age; treatment with oral prednisolone did not alter clinical outcomes or AST concentrations. In GM1‐gangiosidosis cats, Gray‐Edwards et al. [12] observed elevated baseline CSF and serum AST levels, which normalized following intracranial AAV‐mediated gene therapy, correlating with prolonged survival and attenuated disease progression. Gross et al. [26] also demonstrated elevated CSF AST concentrations in untreated GM1‐gangiosidosis cats, with partial normalization occurring approximately 2 years after intravenous AAV gene therapy. Across studies, CSF (and serum) AST levels were consistently elevated in untreated animals and were shown to be useful indicators of disease severity and therapeutic response.

Tonin et al. optimized fluorometric assays for β‐galactosidase activity in CSF and serum, demonstrating sufficient sensitivity for quantitative analysis in GM1 patients. This represents the first validated CSF assay capable of supporting *GLB1*‐targeted gene therapy development [27]. The sensitivity of the CSF and serum β‐gal activity assays, assay precision, and the effect of preanalytical factors on β‐gal activity was examined. The authors report that the CSF β‐gal activity assay is the first of its kind with sufficient sensitivity to quantitatively measure β‐gal enzyme activity in CSF samples from GM1 patients [28].

## 12 Prognostic Biomarkers in GM1

### 12.1 Ganglioside

#### 12.1.1 Biological Plausibility

Gangliosides are glycolipids synthesized from ceramide, modified in the Golgi, and sialylated to form GM3, a precursor for GM2. GM1 and GM2 gangliosides are abundant in the brain, concentrated in caveolae‐rich plasma membrane domains, where they regulate neuronal differentiation, adhesion, signaling, and inflammation. Plasma lyso‐GM1 and lyso‐GM2, measurable by LC–MS/MS, are typically elevated in affected individuals, with concentrations inversely correlated with age. While sphingolipidosis pathophysiology is well described, the precise mechanisms of neurodegeneration remain unclear [29]. The two differ structurally by the number of sialic acids and specific monosaccharides present. Plasma lyso‐GM1‐gangiosidosis and lyso‐GM2, measurable by LC–MS/MS, are typically elevated in affected individuals. In one study, plasma lyso‐GM1‐gangiosidosis and lyso‐GM2 were detectable in most patients with GM1, with concentrations inversely correlated with age [12, 30]. While the general pathophysiology of sphingolipidoses has been well characterized, the exact mechanisms leading to neurodegeneration remain unclear. Animal studies demonstrate that GM1 accumulation correlates with disease progression. In dogs, CSF GM1 levels increase with age; in feline models, intracranial AAV gene therapy significantly reduces CSF gangliosides and prolongs survival. Cyclodextrin‐based therapies have shown reductions in GM1 accumulation in patient fibroblasts, supporting its role as a pharmacodynamic biomarker [30] [31].

Fluorescent imaging and flow‐cytometric assays have been applied to quantify GM1‐ganglioside accumulation in lymphocytes and fibroblasts from individuals with GM1‐gangliosidosis. Flow cytometry enabled rapid detection of approximately 2.5‐ to 3‐fold differences in GM1‐ganglioside levels using minimal amounts of fresh blood, demonstrating high sensitivity and minimal measurement bias. In fibroblasts, Miglustat treatment decreased intracellular GM1‐ganglioside content, confirming its expected substrate‐reduction effect. GM1‐ganglioside burden correlated strongly with clinical severity, indicating that biochemical measurements reflect disease progression and may aid both diagnosis and therapeutic monitoring [27].

#### 12.1.2 Summary

Measurable increases in GM1‐ganglioside across blood‐derived cells, cerebrospinal fluid, and tissues provide a reliable indicator of lysosomal substrate burden, supporting its use as a biomarker for diagnosis and longitudinal disease assessment. Differences in GM1‐ganglioside accumulation parallel the clinical spectrum of GM1‐gangliosidosis and may help distinguish disease subtypes. Clinical data also show that GM1‐ganglioside levels accurately reflect GLB1 enzymatic deficiency, reinforcing their value for monitoring responses to substrate‐reduction or enzyme‐replacement therapies. Together, these findings establish GM1‐ganglioside quantification as a robust biomarker grounded in the central pathophysiology of GM1‐gangliosidosis.

### 12.2 Glycans

#### 12.2.1 Biological Plausibility

*GLB1* deficiency impairs lysosomal degradation of multiple substrates, including GM1 and GA1 glycolipids as well as N‐linked glycans terminating in β‐linked galactose. As a result, a diverse set of glycan metabolites accumulates, reflecting a broader biochemical disturbance than ganglioside storage alone. Elevated levels of glycan species such as dp5, A2G2, and newly described isobaric glycan series highlight systemic metabolic involvement. Increases in these substrates correlate with clinical severity, supporting their use as biomarkers for disease burden and for evaluating therapeutic efficacy. Lawrence et al. [32] used tandem mass spectrometry combined with glycan reductive isotope labeling to characterize the full spectrum of *GLB1*‐dependent substrates. After confirming accumulation of GM1, GA1, and β‐galactose‐terminated N‐glycans, they identified a novel group of structurally related isobaric glycans that also build up in *GLB1* deficiency. By comparing data from murine models with human brain and urine samples, they demonstrated that *GLB1* loss produces a broader oligosaccharidosis, revealing metabolic signatures beyond ganglioside storage. They proposed that comprehensive profiling of all accumulating β‐galactosidase substrates could enhance diagnostic accuracy and provide sensitive readouts for monitoring therapeutic response in GM1. Kell et al. [33] identified two pentasaccharide biomarkers, H3N2a and H3N2b, which were markedly increased in plasma, CSF, and urine from an affected patient. In a feline GM1 model, only H3N2b was consistently detectable. Following AAV‐mediated gene therapy in both the feline model and the patient, H3N2b levels decreased across CNS, plasma, CSF, and urine and showed a negative correlation with restored β‐galactosidase activity. These findings suggest that H3N2b may serve as a sensitive pharmacodynamic biomarker for evaluating gene therapy efficacy.

### 12.3 Inflammatory Markers

Utz (Jarnes) et al. [34] hypothesized that markers of CNS inflammation would be elevated in patients with the severe infantile GM1 phenotype. Using multiplex analysis of 188 analytes in CSF and serum, they identified 13 candidate biomarkers in CSF and two in serum. Five CSF analytes (ENA‐78, MCP‐1, MIP‐1α, MIP‐1β, and TNFR2) remained persistently elevated in patients with the infantile form, suggesting that neuroinflammatory pathways may contribute to the aggressive clinical presentation and may serve as indicators of disease severity.

### 12.4 Chitotriosidase (CHITO)

#### 12.4.1 Biological Plausibility

Progressive neurodegeneration in lysosomal diseases, including GM1‐gangliosidosis, is increasingly understood to involve significant neuroinflammation. Accumulation of gangliosides within lysosomes promotes macrophage activation and propagation of inflammatory signaling pathways. Chitotriosidase (CHITO), or chitinase‐1, is produced almost exclusively by activated macrophages and is considered part of the innate immune response to chitin‐containing pathogens. Because CHITO reflects macrophage burden and lysosomal stress, it has emerged as an indirect marker of tissue injury in several LDs [35], including monitoring response to therapies. CHITO exists in two major forms: a 50‐kDa enzyme secreted into circulation and a 39‐kDa lysosomal isoform. Elevated levels of these isoforms can indicate heightened macrophage infiltration or activation. For more than a decade, CHITO activity in plasma has been used clinically—most notably in Gaucher disease—to monitor inflammation and therapeutic response, where enzyme replacement reduces CHITO levels and treatment interruption results in rebound activity. However, inter‐individual variability complicates interpretation. Approximately 8% of the population carries a 24‐bp duplication in CHIT1, resulting in absent or markedly reduced CHITO activity, which limits its utility as a universal biomarker. Therefore, CHIT1 genotyping is essential before using CHITO as a disease marker in GM1‐gangliosidosis or other LDs [36]. Initial work evaluating CHITO in GM1 has shown significantly elevated CSF levels in patients with the infantile form, supporting the concept that CHITO reflects neuroinflammation in this context. Still, the mechanistic relationship between macrophage activation and disease pathology in GM1 remains incompletely defined.

Wajner et al. [37] analyzed plasma CHITO activity across several lysosomal diseases—including Gaucher, GM1‐gangliosidosis, and Krabbe disease—compared results to healthy controls. Enzyme activity was elevated approximately 600, 15, and 12‐fold, respectively, in these disorders relative to controls. Kinetic parameters (*V*_max_, pH optimum, thermal stability) were also significantly altered in patient samples. Although these findings support potential diagnostic value, CHIT1 polymorphisms must be considered, as CHITO deficiency undermines diagnostic reliability. A case report further highlighted the complexity of metabolic disorders when an infant presenting with developmental delay and metabolic abnormalities was ultimately diagnosed with both dihydropyrimidine dehydrogenase deficiency and GM1‐gangliosidosis. Elevated CHITO activity accompanied severe β‐galactosidase deficiency, illustrating how secondary biomarkers such as CHITO may contribute to recognition of lysosomal involvement even in atypical presentations. In a report of an 8‐month‐old girl presenting with developmental delay, decreased muscle tone, dull affect, and fatigue, a marked excretion of thymine with significantly increased uracil excretion in the urine was observed. The indication of pyrimidine catabolic disorder, that is, dihydropyrimidine dehydrogenase deficiency, was confirmed by endogenous purines. Subsequent purine variant analysis confirmed complete dihydropyrimidine dehydrogenase deficiency. MRI displayed severe hypomyelination with gliosis involving basal ganglia [38]. Within 7 months, she was hospitalized for aspiration pneumonia and seizures, and hepatosplenomegaly, at which time a marked deficiency of β‐galactosidase activity in leukocytes and an elevated CHITO activity indicated GM1. The dual diagnosis of dihydropyrimidine dehydrogenase deficiency and GM1‐gangliosidosis highlights the interconnectedness of inborn metabolic errors.

A retrospective study (*n* = 22) from Germany and Austria documented subtype‐specific patterns. Infantile patients demonstrated classic somatic features, whereas late‐infantile and juvenile forms showed more variable neurological signs. CHITO activity was elevated in 12 of 15 patients tested, and urinary oligosaccharides were abnormal in most cases with available data. Genetic analysis identified 23 pathogenic or likely pathogenic *GLB1* variants, including seven previously unreported variants [2].

The NIH‐funded natural history study by Jarnes et al. [4, 5, 36, 39], evaluated CHITO concentrations in CSF and serum from individuals with neuronopathic lysosomal diseases. CHITO levels were highest in GM1‐gangliosidosis compared with other disorders and were significantly greater in infantile versus juvenile forms [36]. CHITO concentration inversely correlated with cognitive performance, and longitudinal sampling showed increasing CSF CHITO over time in infantile GM1, indicating that CHITO may reflect disease progression. Additional studies are required to validate CHITO as a clinical trial biomarker.

#### 12.4.2 Summary

CHITO shows promise as a diagnostic and monitoring biomarker in GM1‐gangliosidosis, particularly, for capturing neuroinflammatory activity and tracking therapeutic response. However, its usefulness is limited by substantial biological variability, most importantly by CHIT1 polymorphisms causing partial or complete CHITO deficiency. Because GM1‐gangliosidosis itself does not influence CHIT1 genotype, CHITO cannot be reliably interpreted in affected individuals carrying this variant. Prescreening for CHIT1 status is, therefore, essential when considering CHITO for clinical assessments or trial endpoints. At present, standardized guidelines for integrating CHITO into routine diagnostics or therapeutic monitoring have not been established.

### 12.5 Lysosphingolipids (LysoSLs)

#### 12.5.1 Biological Plausibility

In GM1 gangliosidosis, impaired sphingolipid hydrolase activity leads to the accumulation of N‐acylated sphingolipids and a corresponding increase in their N‐deacylated derivatives, LysoSLs [40, 41]. Circulating LysoSLs (e.g., glucosylsphingosine [GlcSph] and globotriaosylsphingosine [lysoGb3]) have been established as sensitive biomarkers across multiple sphingolipidoses, including Gaucher, Fabry, Krabbe, Niemann–Pick disease, and the GM1/GM2 gangliosidoses. In addition to serving as biomarkers, LysoSLs may contribute directly to pathogenesis; this role is best defined in Krabbe disease, where lyso‐psychosine demonstrates potent cytotoxic and pro‐inflammatory effects [41].

Several analytical platforms have been developed to quantify LysoSLs in biofluids. Polo et al. [41] developed a liquid chromatography–tandem mass spectrometry (LC–MS/MS) assay capable of simultaneously measuring multiple LysoSLs (HexSph, lysoGb3, lysoGM1, lysoGM2, lysoSM, and lysoSM509) in DBSs. Using this method, lysoGM1 was markedly elevated in a patient with infantile‐onset GM1 gangliosidosis and was correctly detected in the DBS sample. In a separate study, plasma LysoSLs and protein biomarkers were evaluated in 13 individuals with GM1 gangliosidosis and compared with 66 controls. LysoGM1 was measurable in 12 of 13 patients and undetectable in all controls, reinforcing its disease specificity. Despite these findings and strong preclinical support, clinical validation of lysoGM1 remains limited. In particular, there is little evidence from longitudinal studies, constraining the ability to assess treatment responsiveness or correlate LysoSL dynamics with clinical outcomes [42].

#### 12.5.2 Summary

LysoSLs represent biochemically plausible biomarkers of disrupted sphingolipid metabolism in GM1 gangliosidosis, with lysoGM1 directly reflecting impaired GM1 ganglioside degradation. Beyond accumulation, LysoSLs may exacerbate disease through pro‐inflammatory and cytotoxic pathways. Although gene‐therapy–treated patients in the Passage Bio clinical program (out‐licensed to GemmaBio in August 2024) demonstrated normalization of cerebrospinal fluid LysoSLs alongside early signals of clinical benefit, the overall evidence base for LysoSLs, particularly, lysoGM1, as robust clinical biomarkers remains preliminary.

### 12.6 Lactosylceramide (LacCer)

#### 12.6.1 Biological Plausibility

Skeletal involvement is a recognized feature of GM1 gangliosidosis, and comparative radiologic studies highlight substantial differences between late‐infantile and juvenile disease. In a cohort of 13 late‐infantile and 21 juvenile patients, the average interval from symptom onset to diagnosis was 1.9 years and 6.3 years, respectively [43]. All late‐infantile patients demonstrated odontoid hypoplasia and characteristic pear‐shaped vertebral bodies. Although juvenile patients lacked these classic abnormalities, approximately three‐quarters exhibited irregular vertebral endplates, and nearly half showed central endplate indentation and flattened or squared vertebral morphology. Dual‐energy X‐ray absorptiometry revealed significant reductions in bone mineral density (BMD) at the lumbar spine, femoral neck, and total hip, with lumbar BMD reaching a peak around age 19 and distal forearm BMD peaking by age 30. Despite low BMD across the cohort, no participants had experienced fractures. These findings highlight the presence of early skeletal abnormalities in GM1 and underscore the importance of routine monitoring to support long‐term musculoskeletal health. LacCer has been implicated in the regulation of osteoclastogenesis and bone remodeling. Work by Fukumoto et al. [44] suggests that LacCer participates in osteoclast differentiation, although the precise mechanisms by which glycolipids influence osteoclast biology remain incompletely defined. Beyond skeletal tissues, LacCer is involved in multiple inflammatory and proliferative signaling pathways. Increased LacCer synthase activity during glial cell proliferation, along with LacCer‐induced production of pro‐inflammatory cytokines and inducible nitric oxide synthase (iNOS), indicates an active role for LacCer in neuroinflammatory signaling. Metabolically, LacCer serves as an obligate precursor in the synthesis of complex GSLs, including GM1 ganglioside. In GM1 gangliosidosis, impaired degradation of GM1 is expected to perturb LacCer turnover, potentially contributing to its accumulation. Evidence from other lysosomal storage disorders supports this concept: elevated LacCer is observed in plasma and tissues from individuals with Gaucher and Fabry diseases, where it correlates with lysosomal dysfunction. These data suggest that LacCer may similarly function as a secondary biomarker in GM1, complementing GM1 ganglioside levels by reflecting broader GSL imbalance and lysosomal stress. A comparative immunohistochemical analysis of LacCer in post‐mortem tissue from individuals with GM1 gangliosidosis, Niemann–Pick C disease, Pompe disease, neuronal ceroid lipofuscinoses, Krabbe disease, and mucopolysaccharidoses demonstrated LacCer accumulation across multiple organs despite normal activity of its primary catabolic enzymes [45]. The pattern of accumulation varied by disorder: neuronal accumulation was particularly prominent in Niemann–Pick C, suggesting disorder‐specific differences in GSL turnover within compromised lysosomal–endosomal systems. In GM1 gangliosidosis, LacCer accumulation was detectable but variably expressed, limiting its interpretability as a standalone marker of disease pathology.

#### 12.6.2 Summary

LacCer is a central intermediate in GSL synthesis, and its metabolism is perturbed in GM1 gangliosidosis due to impaired GM1 degradation. LacCer accumulation reflects underlying lysosomal dysfunction and disturbances in GSL flux—core pathological features of GM1. Its involvement in osteoclastogenesis, glial proliferation, cytokine induction, and inflammatory signaling provides a mechanistic link to systemic manifestations such as skeletal abnormalities. LacCer‐driven disruption of lipid rafts and interference with RANKL‐mediated signaling further implicate it in bone–immune axis dysregulation. Although these pathways are supported by experimental and animal data, clinical evidence directly linking LacCer to skeletal pathology in GM1 remains limited. Overall, LacCer shows potential as a secondary biomarker of disease burden and metabolic imbalance, but its clinical utility is not yet established.

## 13 Biomarkers of Neuronal Damage in GM1

Significant neuronal injury occurs early in GM1 gangliosidosis, especially in pediatric‐onset forms, leading to profound abnormalities in brain structure and signal characteristics compared with typically developing children. These disease‐related changes limit the applicability of standard adult or pediatric neuroimaging atlases, which are essential for automated coregistration and segmentation [46]. Due to the rarity of GM1 gangliosidosis, a dedicated, fully automated segmentation pipeline using a natural‐history–derived atlas has not yet been established. Consequently, manual correction or expert‐guided segmentation remains necessary for accurate volumetric assessment in GM1. This methodological barrier underscores the urgent need for robust, quantifiable biomarkers of neuronal damage to support diagnosis, monitor disease progression, and evaluate treatment response in both natural history studies and interventional trials [17, 18].

### 13.1 Neurofilament Light Chain (NfL)

#### 13.1.1 Biological Plausibility

NfL, a marker of neuronal damage and axonal degeneration, has emerged as a potential biomarker for GM1. It is a well‐characterized biomarker of axonal damage and neurodegeneration. It is released into cerebrospinal fluid and blood following neuroaxonal injury, and elevated circulating NfL levels are consistently observed across numerous neurodegenerative disorders, including Alzheimer's disease, frontotemporal dementia, Parkinson's disease, and multiple sclerosis. The same biological principles apply to GM1 gangliosidosis: lysosomal dysfunction, neuroinflammation, and progressive neuronal loss create conditions that lead to the release of NfL into the bloodstream. Accordingly, elevated plasma NfL is considered biologically plausible as a marker of neuronal injury in GM1.

In the study by Welford et al. [42] biomarkers of neuropathology were measured in 13 patients with GM1 gangliosidosis and compared with 66 controls. Plasma NfL levels were markedly elevated in all GM1 patients, with no overlap between patients and controls, indicating high discriminatory potential. Glial fibrillary acidic protein (GFAP), a marker of astrocyte activation, was also elevated in GM1, though with partial overlap with control values. These findings suggest that both axonal and astroglial injury contribute to the neuropathological profile of GM1. Although NfL has demonstrated utility across a range of neurodegenerative and age‐related diseases, its use in GM1 remains limited by the small number of published studies and the absence of longitudinal data. Without serial measurements, it is difficult to determine how well NfL reflects disease trajectory, therapeutic response, or genotype–phenotype variability in GM1. Despite its sensitivity, NfL is a nonspecific biomarker that does not provide information about the anatomical distribution of neuronal injury. This limitation is particularly relevant in GM1 gangliosidosis, where both gray and white matter structures are affected, but the relative contributions of ganglioside accumulation, inflammation, oxidative stress, and impaired autophagy to regional vulnerability remain poorly understood. Moreover, standardized methodologies for blood NfL measurement and harmonized natural history datasets are not yet available in GM1, constraining broader clinical adoption. Given these gaps, complementary neuroimaging biomarkers are essential. Quantitative imaging techniques capable of mapping structural and microstructural brain changes would provide spatial context for NfL elevations, enable a more comprehensive characterization of disease burden, and enhance the ability to monitor therapeutic effects.

### 13.2 Neuroimaging

#### 13.2.1 Biological Plausibility

GM1 gangliosidosis produces widespread neurodegeneration, reflected in structural and signal abnormalities on neuroimaging. Early symptoms, most commonly impairments in gait and expressive language, correlate with progressive involvement of both white matter and deep gray nuclei [47]. Although MRI is not diagnostic, characteristic findings include diffuse white matter signal abnormalities, delayed myelination, and subtle but reproducible changes in the basal ganglia and thalamus. Numerous studies demonstrate that GM1 is associated with volumetric reductions across the cerebrum and cerebellum, white matter atrophy, demyelination, and metabolic disturbances detectable by magnetic resonance spectroscopy (MRS). These observations support MRI as a promising biomarker modality for tracking disease burden and progression. A consistent pattern reported across natural history studies includes macrocephaly, accelerated intracranial volume expansion, and progressive structural loss, particularly, within the cerebellar cortex and deep gray matter [1, 17, 39, 48].

While total brain volume may appear increased in infancy, driven partly by ventriculomegaly and tissue swelling, regional atrophy is simultaneously detectable in the corpus callosum, caudate, and putamen. Elevated intracranial pressure, measured by lumbar puncture, has also been reported in infantile disease and may reflect early hydrocephalus or impaired CSF turnover [10]. MRS findings show reduced N‐acetylaspartate (NAA) and elevated myo‐inositol, consistent with neuronal loss and gliosis. These metabolic changes correlate with clinical severity scores, suggesting their value as quantitative indicators of disease progression. Imaging and EEG from a cohort of GM1 and GM2 patients revealed broad neurodevelopmental regression across infantile and juvenile subtypes. Most children achieved age‐appropriate developmental milestones before symptom onset. Once symptoms emerged, motor regression occurred in 100%, cognitive decline in ~97%, and seizures in ~75%. MRI frequently demonstrated bilateral thalamic abnormalities, brain atrophy, periventricular leukomalacia (PVL), and delayed myelination. EEG often showed low‐voltage backgrounds with epileptiform discharges.

##### 13.2.1.1 Type 1

Infantile onset presents with rapid neuroregression and multisystem involvement. Typical imaging features include basal ganglia hyperintensities, particularly, in the putamen; thalamic hypointensity on T2‐weighted MRI; diffuse white matter hyperintensities and delayed myelination; global cerebral atrophy, which can progress rapidly. Case reports describe severe white matter destruction leading to bilateral frontal cysts, an uncommon but pathophysiologically consistent feature of advanced demyelination. Additional findings include cherry‐red maculae and hepatosplenomegaly, supportive of lysosomal dysfunction [49]. CT and MRI in other infantile cases show cortical atrophy, deep nuclear involvement, and prominent abnormalities in the globus pallidus. These changes reflect oxidative stress, lysosomal failure, and secondary inflammatory injury [50].

##### 13.2.1.2 Type 2

Standardized MRI segmentation remains challenging in GM1 due to extensive neuroanatomic distortion. Zoppo et al. [48] evaluated a semi‐automated segmentation protocol in Type II patients, demonstrating high inter‐ and intra‐rater reliability, with better performance in juvenile than late‐infantile patients. Retrospective analyses of mixed GM1/GM2 cohorts showed high rates of bilateral thalamic involvement (≈63%), periventricular hypomyelination in all patients, and frequent seizures and developmental delay [51, 52, 53].

Regier et al. [17] reported progressive cerebellar and cerebral atrophy in both late‐infantile and juvenile patients, with faster decline in the late‐infantile group. Quantitative MRS showed decreased NAA and elevated myo‐inositol, paralleling clinical regression in expressive language and motor function. Kaiyrzhanov et al. [54] expanded the clinical genetic phenotype of the GM1‐gangiosidosis Type 3 reporting parkinsonism over the disease course of 19 years. Brain MRI revealed well‐defined symmetrical high signal intensities in the posterior part of the putamina on T2‐weighted images.

The decade‐long natural history study, D'Souza et al. [18] further documented progressive brain atrophy, most marked in late‐infantile disease, strong correlations between MRS metabolites (NAA, myo‐inositol) and Vineland functional scores, and phenotypic distinctions from Type I (e.g., preserved hearing, absence of cherry‐red spots or organomegaly). These data refine the understanding of GM1 Type II disease trajectories.

##### 13.2.1.3 Type 3

Adult‐onset GM1 is characterized by parkinsonism, dystonia, and slowly progressive motor impairment. Neuroimaging frequently reveals T2 hypointensity in the putamen and globus pallidus, iron deposition producing the characteristic “wishbone pattern,” and selective neuronal loss in the caudate, putamen, globus pallidus, and cerebellar Purkinje cells [55]. DaT‐SPECT studies show presynaptic dopaminergic dysfunction, supporting clinical parkinsonian features. Proton MRS demonstrates reduced NAA and increased myo‐inositol, suggesting axonal depletion and reactive gliosis. Histology indicates abnormal myelin folding rather than primary axonal destruction, consistent with disrupted axon–glial interactions [50]. The combination of the putamina finding on T2W and the wishbone pattern of iron deposition was also observed by Agarwal et al., suggesting a potential biomarker for type 3 GM1.

Brain CT, MRI, and MR spectroscopy (MRS) findings in a 17‐month‐old Turkish girl with infantile GM1‐gangliosidosis revealed thalamic involvement and a decreased NAA/Cr ratio and an increased Cho/Cr ratio [56]. In a 4‐year‐old boy with neuroregression and optic atrophy, periventricular hyperintensity on MRI revealed white matter changes, as well as beta‐galactosidase enzyme activity being low, which was confirmed by *GLB1* sequencing [57]. Similarly, an 11‐year‐old Japanese girl with Type 3 GM1‐gangliosidosis presenting with clumsiness since early infancy and dystonia since early childhood, which progressed slowly without mental deterioration and dysmorphism, underwent T2‐weighted MRI [58]. Bilateral symmetrical hypointensity in the putamen and globus pallidus was observed by MRI, while CT showed bilateral hyperperfusion in the basal ganglia, which decreased gradually during 1 year of observation. In addition, in a GM1‐gangliosidosis patient with dysmorphism, dysostosis, and progressive dystonia, MRI revealed T2 hypointensity in the basal ganglia [59]. Moreover, in a 66‐year‐old Japanese man with adult GM1‐gangliosidosis, neuronal loss and intracytoplasmic storage were prominent in the caudate nucleus and putamen, as well as (albeit less prominent) in the amygdala, globus pallidus, and Purkinje cells in the cerebellum. The authors concluded that selective neuronal involvement in the CNS is characteristic of adult GM1, reflecting a more active turnover of GM1‐ganglioside in the affected areas than elsewhere in the CNS [60].

Proton MRS detected a mild reduction of NAA, consistent with relative paucity of axons and neurons and increased levels of myoinositol [61]. This presentation has been linked to white matter changes. Although these findings will have to be further confirmed in more patients with GM1, proton MRS may provide useful end points to assess the efficacy of novel treatments. Histological analysis revealed no significant changes in axons; however, there was evidence of excessive and abnormally folded myelin, a characteristic linked to disrupted axon‐glial interactions.

Marangi et al. [62] highlighted a presynaptic dopaminergic dysfunction pattern in adult GM1‐gangliosidosis, reporting hyperintensities in the bilateral putamen and reduced radiotracer uptake in both basal ganglia by DaT‐SPECT (123I‐Ioflupane). The selective involvement of the basal ganglia was believed to result from the region's higher GM1‐ganglioside turnover.

In the NIH‐funded natural history study by Jarnes et al., Nestrasil et al. [39] performed annual quantitative brain MRIs in 14 patients with GM1 and GM2 (9 infantile, 5 juvenile) yearly and compared volumetric analysis with age‐matched controls with the juvenile phenotype. A consistent pattern of macrocephaly and rapidly increasing intracranial MRI volume in the cerebral cortex as well as other smaller structures and ventricular volume were apparent in all infants. The volume of cerebellar cortex decreased in patients with infantile GM1, and total brain volume was significantly less in juveniles with GM1‐gangliosidosis relative to controls.

In the NIH natural history study, annual MRI of infantile and juvenile GM1/GM2 patients revealed persistent macrocephaly and rapid intracranial volume expansion in infants, reduced total brain volume in juveniles compared with controls, progressive atrophy of cerebellar cortex, and ventricular enlargement tracking disease severity. Lewis et al. [46] applied longitudinal diffusion‐weighted imaging and differential tractography to > 100 scans, demonstrating significant progressive loss of white matter fiber tracts in GM1 and normal increases in tract size and number in neurotypical controls. Thus, diffusion‐based metrics may serve as sensitive markers of evolving white matter pathology.

#### 13.2.2 Summary

GM1 gangliosidosis presents substantial challenges for biomarker development due to small patient populations as well as limited accessibility of CNS tissue and constraints imposed by the blood–brain barrier. Persistent limitations due to heterogeneity in clinical progression and lack of standardized imaging protocols challenge the ability to correlate peripheral biomarkers with central pathology. Nevertheless, neuroimaging provides a powerful window into disease biology. Quantitative MRI, MRS, diffusion imaging, and iron‐sensitive sequences collectively offer structural, metabolic, and microstructural measures of neurodegeneration. When combined with fluid biomarkers (e.g., NfL, GFAP, LysoSLs), imaging holds strong potential to serve as a multidimensional framework for tracking disease burden, stratifying patients, and evaluating treatment response in clinical research and therapeutic development.

## 14 Clinical Evidence for Biomarkers of Neuroinflammation and/or Metabolic Disturbances in GM1‐Gangliosidosis

### 14.1 Aspartate Aminotransferase (AST)

#### 14.1.1 Biological Plausibility

In GM1, the accumulation of undegraded GM1 ganglioside induces cellular stress, lysosomal overload, and mitochondrial dysfunction. These processes can damage hepatocytes, leading to the release of AST. Because AST is abundant in both the cytosol and mitochondria, its elevation may reflect mitochondrial stress in addition to hepatocellular injury. Clinical reports describe increased AST levels in GM1 (approximately two to three fold [Jarnes, unpublished data]), particularly, in the infantile subtype, suggesting that AST may index systemic metabolic disruption secondary to lysosomal pathology.

Kilic et al. [25] evaluated biochemical profiles in patients with GM1 or GM2 following enzyme and/or molecular confirmation. AST was elevated in every patient tested, whereas alanine aminotransferase (ALT) remained normal. These findings suggest that isolated AST elevation may serve as a simple but informative biochemical clue in suspected infantile gangliosidosis, with potential utility for diagnosis, prognosis, and monitoring future therapeutics.

A case report by Lan et al. [63] described a young girl with progressive motor decline, limb hypertonia, and frequent subclinical seizure activity. Laboratory evaluations revealed elevated AST, while MRI and diffusion tensor imaging demonstrated widespread white matter abnormalities. Genetic testing identified compound heterozygous *GLB1* variants, including a previously unreported variant, linking AST abnormalities with confirmed GM1‐associated pathology.

In another series involving eight children with developmental regression and refractory epilepsy, WES combined with β‐galactosidase enzyme assays identified *GLB1* variants in all patients. The most common variant, p.D448V, accounted for half of all alleles. Every patient exhibited isolated AST elevation despite normal ALT levels, reinforcing the potential of AST as an ancillary biomarker alongside genetic and imaging assessments [24].

#### 14.1.2 Summary

Although AST is not disease‐specific, isolated AST elevation with normal ALT may strengthen diagnostic confidence in GM1 when combined with clinical features and molecular testing. Its correlation with systemic involvement suggests value as a supportive biomarker of metabolic stress, lysosomal dysfunction, and treatment response.

## 15 Clinical Evidence for Linking Genotype–Phenotype With Biomarkers in GM1

### 15.1 Biological Plausibility

Variants in *GLB1* influence the residual activity of β‐galactosidase and thereby modulate the extent and pattern of GM1 ganglioside accumulation. Severe, typically infantile‐onset variants often produce near‐complete loss of enzymatic activity and more pronounced biochemical perturbations, whereas milder variants correlate with attenuated substrate accumulation. Secondary biomarkers (e.g., NEU1, CTSA, and GM1‐derived metabolites) may reflect the degree of disruption of the lysosomal multienzyme complex and the broader metabolic consequences of particular variants. This framework provides mechanistic justification for integrating genetic data with biomarker profiles to inform diagnosis, prognostication, and therapeutic stratification.

A Brazilian cohort of 65 GM1 patients identified 17 distinct *GLB1* variants, with c.1622–1627insG accounting for half of all alleles. This variant was significantly associated with symptom onset before 1 year of age, highlighting population‐specific genotype–phenotype patterns. Cognitive impairment (82%) and hepatosplenomegaly (56%) were common, underscoring the systemic impact of severe genotypes [64].

In another study of 16 patients aged 3–30 years, dystonia, parkinsonian features, and facial grimacing were correlated with underlying *GLB1* variants. Variation in clinical severity and age at onset corresponded to predicted differences in residual β‐galactosidase activity, emphasizing the role of allelic heterogeneity in shaping phenotype [60]. Structural modeling studies of mutant β‐galactosidase proteins demonstrated that the magnitude of conformational disruption correlates with biochemical and clinical severity. Changes in solvent‐accessible surface area and root‐mean‐square deviation predicted loss of function across representative variants. These structural insights highlight the potential of in silico modeling as a complementary tool in biomarker discovery and mechanistic interpretation.

Because *GLB1* produces two transcripts encoding β‐galactosidase and elastin‐binding protein (EBP), variants may differentially affect these proteins. Caciotti et al. [65] developed quantitative assays to distinguish transcript alterations, identifying multiple novel variants and enhancing genotype–phenotype resolution. EBP dysfunction helps explain cardiomyopathy and connective‐tissue abnormalities in some GM1 patients, further supporting transcript‐level analysis as a relevant biomarker strategy.

### 15.2 Summary

Genotype–phenotype correlation research in GM1 enhances biomarker discovery by linking specific variants to substrate accumulation patterns, disease severity, and clinical variability. Genotype‐stratified biomarker profiling can facilitate personalized diagnostic approaches and may guide the development of variant‐specific therapeutic strategies.

## 16 Biomarkers Lacking Clinical Evidence

### 16.1 Cathepsin A (CTSA) and Neuraminidase (NEU1)

GM1 shares biochemical overlap with Morquio B, galactosialidosis, and sialidosis, suggesting potential roles for CTSA and NEU1 as biomarkers. CTSA stabilizes the lysosomal multienzyme complex comprising β‐galactosidase and NEU1. In GM1, *GLB1* variants lead to β‐galactosidase deficiency, potentially destabilizing this complex and secondarily altering CTSA activity. NEU1, responsible for removing sialic acid residues from glycoconjugates, may also be indirectly affected by the dysfunctional enzyme complex [19]. Although the mechanistic basis for altered CTSA or NEU1 activity in GM1 is biologically plausible, current evidence is indirect and largely inferred from related lysosomal storage disorders. No studies have yet demonstrated that enzyme correlates with GM1 disease severity or treatment response. Thus, validation in patient‐derived samples is essential before considering CTSA and NEU1 as clinically actionable biomarkers [66].

NEU1 catalyzes the removal of sialic acid residues from glycoproteins and glycolipids, processes that are disrupted in GM1. The functional association with β‐galactosidase and its involvement in lysosomal homeostasis in GM1‐gangliosidosis provides a scientific rationale for exploration. NEU1 forms a functional complex with β‐galactosidase and CTSA [67]. In GM1, β‐gal deficiency destabilizes this complex, potentially altering NEU1 levels or activity. NEU1 activity may be indirectly affected by this dysregulation [66], making it a potential marker of lysosomal stress. It has been speculated that restoring lysosomal function via ERT or gene therapy may normalize NEU1 activity, making it a candidate for assessing therapeutic efficacy [67]. Thus, current evidence for CTSA and/or NEU as biomarkers in GM1‐gangliosidosis is indirect and extrapolated from studies in related diseases. Validation studies are necessary to determine whether CTSA levels or activity correlate specifically with disease progression or therapeutic response in GM1‐gangliosidosis patients. Comprehensive profiling in patient‐derived samples (e.g., plasma, CSF) is warranted to clarify its utility and specificity as a biomarker.

### 16.2 Osteopontin and IGFBP‐1

Through quantification of analytes in CSF and serum from living human patients with longitudinal (serial) measurements, Jarnes‐Utz et al. [34] analyzed CSF and serum from serially evaluated patients with gangliosidosis and identified two serum analytes, osteopontin and IGFBP‐2, and five CSF inflammatory mediators consistently elevated in infantile GM1: ENA‐78, MCP‐1, MIP‐1α, MIP‐1β, TNFR2. These analytes differentiated gangliosidosis from other neurodegenerative lysosomal disorders and tracked with clinical severity, suggesting they may reflect CNS‐specific neuroinflammatory activity.

A series of computational methods examining the variants in *GLB1* protein was initially performed with 689 amino acid variants for a sequence‐based screening in silico [68]. Subsequently, structure‐based analysis was performed to understand the interacting profile of the native protein and variants with miglustat. The identification of a native (3THC) structure and two variant structures as well as the description of variantal activity facilitated a more comprehensive understanding of the most deleterious mutants and the miglustat activity on the protein structure providing insight on the stability of the drug with the native and selected variants [68].

### 16.3 Ceramide Tetrahexoside

Suzuki et al. [69] examined ceramide species in GM1 brain tissue, demonstrating that ceramide tetrahexoside predominates within the ceramide hexoside fraction, whereas ceramide trihexoside is present only in small amounts. Gray matter contained elevated glucocerebroside and ceramide lactoside (ceramide dihexoside). These abnormalities suggest profound downstream disturbances in GSL flux and energy metabolism, particularly, in gray matter.

## 17 Proteomics

The identification of low‐molecular‐mass biomarkers via untargeted, multianalyte approach involving high‐resolution 1H NMR analysis has been described by several laboratories. Percival et al. [70] employed high‐resolution ^1^H NMR spectroscopy with multivariate statistical analysis in plasma from 10 Type II GM1 patients. The results revealed upregulation of seven amino acids, downregulation of alanine and lipoprotein‐associated triacylglycerols, and broad disturbances across branched‐chain amino acid degradation, propanoate metabolism, ethanol and amino‐sugar pathways, seleno‐amino acid metabolism, glutathione pathways, alanine metabolism, and fatty acid biosynthesis. A genome‐scale metabolic network model linked these changes to dysfunction in fibroblasts, mitochondria, the Golgi apparatus, and spleen. These findings support substantial systemic metabolic imbalance and align with known mechanisms of neuromuscular dysfunction in GM1.

Table 1 presents a comprehensive summary of candidate biomarkers for GM1. The table integrates biochemical, neuroimaging, inflammatory, metabolic, and genotype‐associated markers, outlining their biological rationale, available clinical evidence, and overall evidentiary strength. Biomarkers with substantial and reproducible data demonstrate the highest potential for assessing disease burden and progression. Others, including LysoSLs, NfL, AST, and metabolomic signatures, show moderate support and serve as promising adjunctive or exploratory indicators. Biomarkers with minimal or indirect evidence, such as CTSA, NEU1, and certain lipid species, require further validation. Together, these data provide a structured framework for prioritizing biomarker development in GM1 and guiding future clinical and translational studies.

**TABLE 1:** Summary of candidate biomarkers for GM1.

Biomarker/biomarker class	Biological rationale	Clinical evidence	Evidentiary strength
LysoGM1 (lysosphingolipid)	Direct product of impaired GM1 ganglioside degradation; reflects lysosomal substrate accumulation	Elevated in GM1 plasma/DBS; detected in nearly all affected patients; limited longitudinal data	Moderate
Other LysoSLs (HexSph, LysoGb3, LysoGM2, LysoSM)	Indicators of secondary glycosphingolipid imbalance	Evidence from small cohorts; not specific to GM1	Low‐moderate
Lactosylceramide (LacCer)	Precursor to GM1; accumulation reflects metabolic imbalance, neuroinflammation, and altered osteoclast signaling	Variable tissue accumulation; inconsistent measurement across studies; implicated in skeletal pathology	Low
Neurofilament light chain (NfL)	Released with axonal injury and neurodegeneration	Elevated in GM1 with no overlap vs. controls; strong biological plausibility; sparse longitudinal data	Moderate
GFAP	Marker of astrocyte activation and neuroinflammation	Elevated in GM1 but overlaps with controls; not disease‐specific	Low‐moderate
MRI Volumetrics (global/cerebellar atrophy, ventricle volume, white matter loss)	Structural and regional markers of neurodegeneration	Strong natural‐history evidence; correlates with clinical severity; reproducible across studies	High
Diffusion MRI/differential tractography	Detects microstructural white matter loss	Longitudinal evidence of progressive tract degeneration unique to GM1	High (emerging modality)
Magnetic resonance spectroscopy (NAA↓)	NAA reflects neuronal integrity; myoinositol reflects gliosis	Consistent abnormalities correlating with severity and trajectory	High
Iron deposition (“wishbone pattern”)	Chronic basal ganglia involvement and iron dysregulation in Type III GM1	Observed in multiple cases; subtype‐specific	Moderate (type III‐specific)
AST (with normal ALT)	Reflects mitochondrial and hepatocellular stress secondary to lysosomal overload	Consistently elevated across several cohorts; not specific to GM1	Low‐moderate (adjunctive)
Ceramide tetrahexoside/dihexosides	Indicate downstream glycosphingolipid dysregulation in gray matter	Tissue‐level evidence only; not validated clinically	Low
Osteopontin (serum)	Marker of chronic inflammation; elevated in gangliosidosis	Elevated in serum; correlated with severity in natural‐history cohort	Moderate (emerging)
IGFBP‐2 (serum)	Reflects metabolic stress and neuroinflammation	Persistently elevated in GM1 infantile patients	Moderate (emerging)
CSF cytokines (ENA‐78, MCP‐1, MIP‐1α, MIP‐1β, TNFR2)	Reflect CNS‐specific neuroinflammation and microglial activation	Elevated only in CSF of infantile GM1; distinct from other lysosomal diseases	High (infantile‐specific)
Proteomic/metabolomic signatures (amino acids, lipids)	Capture systemic metabolic dysfunction (BCAA breakdown, fatty acid metabolism, glutathione pathways)	NMR/metabolomics studies show clear GM1‐specific patterns	Moderate (requires validation)
CTSA and NEU1	Components of lysosomal multienzyme complex dysregulated by β‐gal deficiency	Only indirect evidence; no patient‐level validation	Low
Genotype‐linked biomarkers (variant‐specific substrate patterns, β‐Gal residual activity)	Variants produce predictable biochemical severity; support personalized biomarker profiles	Strong genotype–phenotype associations; robust population evidence (e.g., c.1622–1627insG)	High (for stratification, not stand‐alone biomarkers)

## 18 Discussion

Despite significant advances in understanding the molecular basis and natural history of GM1‐gangliosidosis, no disease‐modifying therapies currently exist that can halt or reverse the progressive neurodegeneration characteristic of this disorder. Current management remains largely supportive, focusing on symptom control, maintenance of function, and quality‐of‐life preservation. Supportive interventions play critical roles in mitigating morbidity, yet access to these services often varies across geographic regions and socioeconomic groups, underscoring the inequities faced by many affected families. Delayed or missed diagnosis remains a major obstacle in GM1‐gangliosidosis. The broad phenotypic spectrum combined with overlapping features of other lysosomal storage and neurodegenerative diseases contributes to diagnostic latency. The lack of standardized NBS programs and limited availability of enzymatic or molecular diagnostic platforms further hinder early detection. Earlier diagnosis would not only enable optimized supportive care but also improve patient eligibility for emerging disease‐modifying trials. Comprehensive natural history studies are still limited, particularly, those that longitudinally characterize multisystem involvement and the quantitative trajectory of neurodegeneration. Understanding genotype–phenotype correlations and their relationship to biochemical and imaging biomarkers is essential to delineate disease heterogeneity and identify predictors of rapid progression versus attenuated forms.

A central unmet need in GM1‐gangliosidosis is the development of validated biomarkers capable of reflecting disease activity, progression, and treatment response. Biomarkers such as lyso‐GM1, NAA (MRS), NfL, chitotriosidase, and inflammatory cytokines have demonstrated preliminary utility across preclinical and clinical studies. However, few have undergone analytical validation or qualification for clinical use. Integrating fluid biomarkers with advanced neuroimaging metrics (e.g., volumetric MRI, diffusion‐weighted imaging, myo‐inositol quantification) may enable multidimensional assessments of CNS pathology and therapeutic efficacy. These tools could be instrumental in designing adaptive clinical trials, monitoring treatment impact, and serving as surrogate endpoints in regulatory pathways. Multiple therapeutic strategies are under active investigation, including AAV‐mediated gene therapy, substrate reduction therapy, pharmacological chaperones, and enzyme replacement approaches. While early data in canine and feline models demonstrate encouraging restoration of β‐galactosidase activity and reduction of lysosomal substrate accumulation, translation to humans requires rigorous assessment of dose safety, biodistribution, and immune responses. Additionally, variability in residual enzyme activity and genotype‐specific molecular effects suggest that patient stratification based on genetic background and biomarker signatures will be essential to optimize therapeutic responses. Longitudinal biomarker‐based endpoints may further elucidate treatment durability and inform dosing paradigms. The inclusion of genotype‐stratified biomarker analyses in future natural history and interventional studies will strengthen the translational continuum from bench to bedside.

In sum, GM1‐gangliosidosis remains a devastating and under‐diagnosed lysosomal disorder with limited therapeutic options. Advancing patient outcomes requires a multifaceted approach encompassing earlier detection, genotype‐specific disease modeling, and biomarker‐driven clinical trial designs. Collaboration across research consortia, industry sponsors, and regulatory bodies will be critical to establishing standardized biomarker frameworks and validated clinical outcome measures. These efforts will not only accelerate the development of effective therapies but also transform clinical management paradigms for patients and families affected by GM1‐gangliosidosis.

## Funding

The authors have nothing to report.

## Conflicts of Interest

The authors declare no conflicts of interest.

## Data Availability

Data sharing not applicable to this article as no datasets were generated or analysed during the current study.

## Appendix A

### Current Treatment Options

Currently, there is no cure for GM1. Treatment is focused on managing symptoms and providing supportive care. In GM1, treatment involves physical therapy, occupational therapy, and speech therapy to help individuals with developmental delays and muscle weakness. In some cases, medications may be used to control seizures and other symptoms.

Beyond palliative, symptomatic and supportive therapeutic approaches, no FDA‐approved treatments exist for GM1‐gangliosidosis patients. Researchers are critically evaluating the efficacy of substrate reduction therapy (SRT), pharmacological chaperones, enzyme replacement therapy (ERT), stem cell transplantation, and gene therapy for GM1. It has been reported that Miglustat (butyl‐deoxynojirimycin, NB‐DNJ) may slow disease progression in patients with juvenile and adult GM1‐gangliosidosis by acting as a substrate inhibitor compound.

### ERT

Current ERT relies on receptor‐mediated endocytosis, which limits effectiveness in hard‐to‐treat organs, including those critical for GM1‐gangliosidosis. In this study, plant‐derived fusion proteins combining β‐galactosidase (β‐gal) and the RTB lectin demonstrated successful uptake via adsorptive‐mediated endocytosis, substrate reduction, and lysosomal activation in GM1‐gangliosidosis patient fibroblasts. Unlike plant‐derived β‐gal alone, β‐gal:RTB fusions supported greater enzyme accumulation and activity compared to traditional mannose‐6‐phosphate‐based delivery mechanisms. These results highlight the potential of lectin‐based strategies to enhance biodistribution and uptake of lysosomal disease therapeutics, offering promising new avenues for treatment [71].

### Chaperone Therapy

Chaperone therapy, based on early molecular studies, led to the identification of galactose and other active‐site binding substrate analogs that stabilized and enhanced the mutant enzyme activity in culture cells. Suzuki et al. reported the mutant misfolding enzyme protein and substrate analog competitive inhibitor to restore the catalytic activity of the mutant enzyme to develop a commercially available compound for Fabry disease, reaching phase 3 development [72]. Subsequently, they found two valienamine derivatives, N‐octyl‐4‐epi‐β‐valienamine (NOEV) and N‐octyl‐β‐valienamine (NOV), as promising therapeutic agents for GM1. Additionally, a commercial expectorant drug, ambroxol, was found to be a chaperone for β‐glucosidase, with a small group of Gaucher patients responding with remarkable improvement of oculomotor dysfunction and myoclonus and providing a potential opportunity for chaperone therapy availability in GM1‐gangliosidosis patients with CNS involvement.

### SRT

Two studies in Italy evaluated SRT with oral Miglustat. The study by Deodata et al. was completed in 2017 (*n* = 3). Deodato et al. [73] first reported positive results of Miglustat (OGT 918, *N*‐butyl‐deoxynojirimycin) treatment in two patients with the juvenile form and one with adult form, started at the age of 10, 17, and 28 years; age at last evaluation was 21, 20, and 38, respectively. Response to treatment was evaluated using neurological examinations ~4–6 months. The baseline neurological status was severely impaired, with loss of autonomous ambulation and speech in the juvenile patients, and gait and language difficulties in the adult patient. All three patients showed gradual improvement while being treated; both juvenile patients regained the ability to walk without assistance for a few meters, and increased alertness and vocalization, suggesting that Miglustat may help slow down or reverse the disease progression in juvenile/adult GM1. In a second Miglustat SRT study (*n* = 4), Fischetto et al. reported improved kyphosis, decreased motor and language skills in one patient, stable impairment in one patient and severe decrease in cognitive, motor and attention abilities in two patients. Miglustat was also tested in the United States (NCT02030015) for infantile GM1, although ultimately unsuccessful, with death in 13 of 16 patients prior to termination in 2021. The Italian clinical trials have demonstrated the potential of SRT in seven patients, but SRT cannot degrade previously accumulated substrate, making early diagnosis and intervention significant.

### Stem Cell Therapy

For GM1, the leading approach utilizes viral vectors, predominantly adeno‐associated viruses (AAVs) for the delivery of functional DNA to target cells, to restore β‐gal activity to its normal state with a single injection. The use of hematopoietic stem cell transplantation (HSCT) to replace defective enzymes and gene editing techniques to correct variants ex vivo before transplantation are also under investigation. Emerging applications explore induced pluripotent stem cells (iPSCs) to develop enzyme‐replacement platforms or enhance neural regeneration. However, challenges include immune rejection, incomplete enzymatic correction across tissues, ethical concerns, and the need for improved delivery to the central nervous system. Clinical trials are ongoing to address these obstacles and optimize outcomes.

- *AAV*: AAV vectors have emerged as a leading platform for delivering therapeutic genes due to their safety profile and ability to transduce various cell types, including neurons. A Phase I/II clinical trial for GM1‐gangliosidosis children is ongoing to evaluate the safety and efficacy of AAS‐mediated *GLB1* delivery by intravenous injection [11]. A second, evaluating AAVhu68 viral vector is also on‐going [74].
- *HSCT*: Two studies evaluated the effectiveness of HCST. NCT00176904, completed in 2010, reported on 135 patients, 120 reached the 100‐day mark, 92 reached the 1‐year mark, and 81 reached the 3‐year mark. In a second, NCT01626092 completed in 2013, 1/3 patients achieved engraftment at the 100‐day mark. Other clinical trials have been initiated in recent years but terminated before completion, including a gene therapy trial (NCT04273269) and another testing umbilical cord‐supported stem cell therapy (NCT00654433).
- *Patient‐derived induced pluripotent stem cells (iPSCs)*: iPSCs have been investigated as cellular models of GM1‐gangliosidosis as potential therapeutic agents for future clinical applications. The generation of iPSCs derived from patients with GM1 produced neurons displaying impaired neurotransmitter release and disturbed presynaptic function related to accumulation of GM1#ganglioside. A high‐content drug‐screening system was then established and identified two compounds as drug candidates which were shown to activate autophagy pathways, reducing GM1‐ganglioside accumulation in vitro and in vivo, and restoring the presynaptic dysfunction [75].
- *PS Gene‐editing platform*: Utilization of CRISPR to introduce a synthetic β‐galactosidase gene into the albumin n locus has been shown to be a potent method of providing a development of the PS Gene‐editing platform.

In summary, ERT, SRT, stem cell therapy, and gene editing are promising treatments for GM1; however, effectiveness is limited for neuropathic GM1‐gangliosidosis due to the restrictive nature of the blood–brain barrier (BBB). ERT and SRT alleviate substrate accumulation through exogenous supplementation over the patient's lifetime, while gene editing could be curative, fixing the causative gene, *GLB1*, to enable endogenous enzyme activity. Stem cell therapy can be a combination of both, with ex vivo gene editing of cells to cause the production of enzymes. These approaches require special considerations for brain delivery, which has led to novel formulations [76]. A few therapeutic interventions have progressed to early‐phase clinical trials, presenting a bright outlook for improved clinical management for GM1. Gene therapy approaches may be high risk–high reward options for GM1‐gangliosidosis patients, capable of correcting the disease if treatment is done early.
